# A Multidimensional Assessment of the Impact of Healthcare Volunteering in a War-Affected Setting

**DOI:** 10.3390/ejihpe16090144

**Published:** 2026-09-19

**Authors:** Latefa Ali Dardas, Amal Abuabada, Amjad Al-Khayat, Firas Al-Mahasneh, Belal Aldabbour

**Affiliations:** 1 Deanship of Scientific Research, The University of Jordan, Amman 11942, Jordan; 2 Gaza Community Mental Health Program, Gaza P.O. Box 1049, Palestine; 3 Department of Educational Sciences, Salt Faculty, Al-Balqa’ Applied University, Al-Salt 19117, Jordan; 4 Center of Excellence in Education and Learning, The University of Jordan, Amman 11942, Jordan; 5 Faculty of Medicine, Islamic University of Gaza, Gaza P.O. Box 108, Palestine

**Keywords:** medical students, volunteering impact, war, conflict settings, Gaza, humanitarian healthcare, mental health, wellbeing

## Abstract

**Background**: Medical students who volunteer during armed conflict may help sustain overwhelmed health services while experiencing concurrent social, academic, psychological, and physical consequences. However, no context-sensitive instrument has been available to assess these multidimensional benefits and burdens in active conflict settings. This study primarily aimed to develop and conduct an initial psychometric evaluation of the Volunteering Impact Scale (VIS) among medical students volunteering in Gaza and, secondarily, to characterize their volunteering experiences. **Methods:** A cross-sectional survey was conducted among 169 medical students who volunteered in hospitals in Gaza during the ongoing war. An initial 28-item pool was generated from a focused literature review, relevant theoretical frameworks, and consultations with Gaza-based medical students and clinicians. A multidisciplinary team reviewed the items for relevance, clarity, redundancy, and contextual appropriateness. Following item refinement and psychometric evaluation, the final 16-item VIS comprised five domains: social impact, mental burden, physical burden, academic development, and wellbeing and fulfillment. Its structure was assessed using exploratory factor analysis with principal axis factoring and Promax rotation, followed by confirmatory factor analysis. Internal consistency and convergent and discriminant validity were also examined. Domain scores and their associations with participant, volunteering, and war-exposure characteristics were subsequently analyzed. **Results**: Exploratory factor analysis revealed a five-factor structure explaining 51.2% of the variance. Parallel analysis favored three factors, whereas a theoretically informed five-domain solution was retained for further evaluation. Primary factor loadings ranged from 0.23 to 0.89. Most items met the prespecified loading criterion of 0.40; however, three Social items demonstrated weaker loadings and were retained provisionally because of their conceptual relevance to the construct. Confirmatory analysis provided support for the proposed measurement structure (RMSEA = 0.059, SRMR = 0.075, CFI = 0.911, and TLI = 0.888). Cronbach’s alpha coefficients ranged from 0.64 to 0.75, and discriminant validity was supported by heterotrait–monotrait ratios below 0.85. Participants reported perceived social (M = 4.31/5), academic (M = 4.19), and wellbeing-related (M = 3.96) benefits. Mean reverse-scored Mental and Physical scores were 2.71 and 2.60, respectively, with lower scores indicating greater burden. Longer volunteering was associated with greater physical strain but also with stronger academic and wellbeing outcomes. **Conclusions**: The VIS demonstrated a conceptually coherent five-domain structure and promising initial psychometric properties for assessing the concurrent benefits and burdens of healthcare volunteering in a war-affected setting. Further validation in independent and more diverse samples is required. The findings also indicate that volunteer programs should combine meaningful clinical participation with structured supervision, recovery time, role rotation, and context-sensitive psychosocial support.

## 1. Introduction

Volunteering is an important platform for civic engagement and personal development. Volunteers contribute their unremunerated time and effort to address unmet needs in individuals and society and support local communities (Strkljevic et al., 2024). Although altruistic in nature, volunteering is a multidimensional phenomenon with broad implications for the individual volunteer (Clary et al., 1998; McCauley et al., 2021; Strkljevic et al., 2024). Observational studies of volunteering in general suggest that volunteers experience reduced depression, greater life satisfaction, and, in some cases, lower mortality rates, effects mediated by enhanced social integration, emotional fulfillment, and meaning-making, positioning volunteering as a potential public health intervention that reinforces personal growth and social capital (Jenkinson et al., 2013).

Medical volunteers, in particular, are uniquely positioned to benefit across multiple domains. The additional clinical experience gained through volunteering has been associated with numerous professional, academic, and social benefits. Medical volunteers across a variety of settings reported improved diagnostic reasoning and practical skills, enhanced communication, increased patient understanding and insight, and greater cultural humility. Volunteers also frequently reported that the experience heightened their appreciation of peers, improved teamwork and leadership skills, supported academic development through real-world exposure, and enhanced overall wellbeing and a sense of fulfillment, including a renewed sense of purpose and professional identity. At an aggregate level, medical volunteering contributes to the transfer of medical knowledge, improves the quality of healthcare providers, and fosters reinforced commitment and workforce resilience (Bazan et al., 2021; Lu et al., 2018; McCauley et al., 2021; Strkljevic et al., 2024).

However, volunteering is also a paradoxical phenomenon that is not universally beneficial. Exposure to suffering, death, resource scarcity, and ethical dilemmas may impose considerable psychological and physical burdens on the volunteer. The literature on medical volunteering highlights an increased risk of emotional exhaustion, burnout, and moral distress. Physically, there is a risk of sleep disturbance and physical strain, and in certain settings, a real risk to personal security. This is particularly relevant for students and early-career volunteers who work beyond their standard roles or training, those who work in settings that lack organizational preparation and structured supervision, and medical volunteers in conflict zones (Dell et al., 2014; Umar et al., 2022). At the collective level, some worry that poorly organized volunteer missions may erode local health strategies rather than support them and create dependencies in the communities they serve (Bauer, 2017; Irfan et al., 2025; Rovers et al., 2016).

Existing instruments capture important but distinct components of volunteering. The 30-item Volunteer Functions Inventory (VFI), one of the most widely used measures in the field, assesses six functions that volunteering may serve: values, understanding, social relationships, career development, psychological protection, and personal enhancement (Clary et al., 1998). The VFI was designed primarily to explain why individuals volunteer and has demonstrated relationships between the fulfillment of volunteers’ motives, satisfaction, and intentions to continue volunteering. It does not, however, provide an integrated assessment of the consequences experienced after volunteering, particularly the simultaneous benefits and burdens that may emerge in high-risk clinical environments. The Volunteer Satisfaction Index (VSI) addresses a different part of the volunteering experience by measuring organizational support, participation efficacy, empowerment, and group integration (Galindo-Kuhn & Guzley, 2001). Although useful for evaluating satisfaction and volunteer retention within organizations, its emphasis is on organizational experience rather than the volunteer’s psychological, physical, academic, and broader wellbeing outcomes. The International Volunteer Impacts Survey (IVIS) represents a more explicit attempt to assess volunteering outcomes. Its development incorporated domains such as international understanding, intercultural relationships, international social capital, social skills, civic engagement, life plans, and career intentions (Lough et al., 2009; McBride et al., 2012). The IVIS therefore offers a broad assessment of the developmental and civic effects of international service. Nevertheless, it was developed for structured international volunteering and emphasizes intercultural and civic outcomes; it does not foreground the immediate physical strain, trauma-related psychological burden, disrupted education, clinical learning, and local social connectedness that may coexist among healthcare students volunteering within their own war-affected communities.

Measurement in emergency and healthcare-student volunteering has remained similarly fragmented. Studies of medical students during the COVID-19 pandemic have commonly focused on willingness and readiness to volunteer, motivations, barriers, competence, logistics, and perceived safety rather than on the multidimensional consequences of completed volunteering (Bazan et al., 2021; Byrne et al., 2023; Umar et al., 2022). Conversely, research involving disaster and humanitarian volunteers has generally assessed specific adverse outcomes—such as post-traumatic stress, depression, burnout, or secondary traumatic stress—using separate symptom-focused instruments (Aldamman et al., 2019; Thormar et al., 2010). These approaches provide valuable information but address different measurement purposes and do not necessarily capture the particular combination of experiences relevant to local medical-student volunteers during armed conflict. The VIS was developed to assess a context-specific combination of perceived social, academic, wellbeing, psychological, and physical consequences of healthcare volunteering among medical students in a war-affected setting. Its intended contribution is to complement, rather than replace, established measures of volunteer motivation, satisfaction, intercultural development, or psychiatric symptoms.

The Gaza Strip has been engulfed in war since 7 October 2023, resulting in massive casualties, widespread destruction, and the collapse of the healthcare system. By the end of 2025, nearly 200,000 casualties have been documented, with repeated attacks on healthcare facilities killing hundreds of healthcare workers and forcing many hospitals to cease operations (Gisha, 2025). The destruction also did not spare the two medical schools operating in the Strip, with detrimental impacts on medical education. Medical students’ experiences should be understood within the broader psychological burden affecting Gaza’s civilian population. An online survey of 405 adults living in Gaza, conducted between November 2024 and January 2025, found that 72.7% screened positive for moderate-to-severe depression, 65.0% for moderate-to-severe anxiety, and 83.5% for probable PTSD (Zughbur et al., 2025). These findings indicate substantial psychological distress in a broader civilian sample, although they should not be interpreted as population-representative prevalence estimates. Separately, a study of 339 medical students in Gaza reported symptoms of at least mild severity in 97.05% for depression, 84.37% for anxiety, and 90.56% for stress; 63.40% met the study’s screening criteria for PTSD (Aldabbour et al., 2024). Differences in sampling, assessment periods, instruments, and symptom thresholds preclude direct comparison between these estimates and those from the civilian sample. Thus, the available evidence does not establish whether medical students experience greater or lesser psychological distress than other Gaza residents. Their VIS responses may reflect both the shared consequences of war exposure and experiences associated with their clinical volunteering roles.

In this context of calamity and scarcity, healthcare student volunteers, including undergraduate medical and nursing students, became essential to sustaining emergency care, supporting mass-casualty responses, and filling critical staffing gaps, all while being part of the traumatized population they served. Frameworks such as Altruism Born of Suffering suggest that such prosocial engagement during crises may promote resilience and post-traumatic growth, whereas trauma-exposure research warns of exacerbated burnout and PTSD (Staub & Vollhardt, 2008; Thormar et al., 2010). These pressures are intensified in collectivist and deprived contexts like Gaza, where strong social cohesion reinforces shared coping and communal obligation, simultaneously deepening resilience while exacting a high psychological cost (Weiss & Berger, 2010).

Against this background, the need for the VIS arises from both a contextual and a measurement gap. Medical students volunteering in Gaza were simultaneously students, healthcare responders, members of the affected community, and individuals directly exposed to war. Existing volunteering instruments were not designed to capture this convergence of clinical learning and social contribution with psychological and physical burden. Accordingly, the primary aim of this study was to develop and conduct an initial psychometric evaluation of the VIS as a multidimensional measure of the social, mental, physical, academic, and wellbeing impacts of hospital-based volunteering in a war-affected setting. The secondary aim was to characterize these impacts among medical students volunteering in Gaza and examine how they varied according to participant characteristics, volunteering exposure, and war-related experiences.

## 2. Methods

### 2.1. Study Design and Setting

This study employed a cross-sectional design. Data were collected between May and July 2025 during the ongoing Gaza war, a period marked by large-scale population displacement, destruction of healthcare infrastructure, and significant limitations on medical education. The study was conducted among medical students who volunteered in hospital settings across the Gaza Strip during the conflict. The methodological approach established best practices for scale development in high-adversity and humanitarian contexts, including item generation grounded in theory and lived experience, exploratory and confirmatory factor analyses, and multiple forms of validity assessment.

### 2.2. Participants and Recruitment

Eligible participants were undergraduate medical students enrolled at either of the two medical schools in the Gaza Strip prior to October 2023, who had engaged in hospital-based volunteering during the war. Volunteering was defined as providing unpaid clinical or clinical support services in hospital wards or emergency departments for a minimum of 4 consecutive weeks during the conflict period. All participants were Palestinian residents of Gaza during the study period. Students who volunteered exclusively in non-clinical community roles were excluded. Participants received no clinical training credits for volunteering because war-related disruptions prevented the administrative arrangements required for formal academic recognition.

Participants were identified through faculty contacts, hospital volunteer rosters, and peer referral networks to ensure coverage of both preclinical and clinical training levels and representation across major hospitals. A total of 233 eligible volunteers were invited to participate via phone and email; 169 completed the survey (response rate 72.5%). This sample size exceeded minimum recommendations for factor analysis, satisfying subject-to-item ratios greater than 5:1 and absolute sample size thresholds commonly cited for stable factor solutions.

### 2.3. Development of the Volunteering Impact Scale

The VIS was developed as a theory-informed, context-sensitive measure of the consequences of healthcare volunteering during armed conflict. It was not derived from a single theoretical model. Instead, its conceptual framework integrated theories explaining positive adaptation and prosocial engagement following adversity with trauma-exposure perspectives describing the potential psychological and physical costs of assisting others in high-risk environments. This approach was selected because the study conceptualized volunteering as an experience that may generate benefits and burdens concurrently rather than as an exclusively positive or negative activity.

Altruism Born of Suffering provided the principal theoretical basis for the positive dimensions of the instrument. This framework proposes that personal or collective exposure to suffering can motivate prosocial action and contribute to meaning, connection, and constructive psychological change (Staub & Vollhardt, 2008). It informed items concerning social connectedness, communal contribution, empowerment, and wellbeing. Trauma-exposure perspectives and research involving disaster and humanitarian volunteers informed the Mental and Physical domains. This literature shows that repeated exposure to death, injury, resource scarcity, sleep disruption, moral dilemmas, and prolonged workload can contribute to distress, helplessness, exhaustion, burnout, and trauma-related symptoms among volunteers (Aldamman et al., 2019; Dell et al., 2014; Thormar et al., 2010; Umar et al., 2022).

The volunteering-measurement literature provided an additional basis for defining the proposed domains. The Volunteer Functions Inventory identifies values, understanding, social, career, protective, and enhancement functions of volunteering (Clary et al., 1998), while the Volunteer Satisfaction Index emphasizes organizational support, participation efficacy, empowerment, and group integration (Galindo-Kuhn & Guzley, 2001). The International Volunteer Impacts Survey extends assessment to social skills, civic engagement, international understanding, social capital, and life and career plans (Lough et al., 2009; McBride et al., 2012). These instruments informed the inclusion of social connection, learning, professional development, perceived support, and personal fulfillment. However, because they principally assess motivation, satisfaction, organizational experience, or international service outcomes, they did not adequately represent the concurrent trauma-related, physical, and educational consequences faced by local medical students volunteering during active conflict. Studies of medical-student volunteering further supported the inclusion of academic development, practical competence, safety, psychological impact, and role-related demands (Bazan et al., 2021; Byrne et al., 2023; Dell et al., 2014; Lu et al., 2018; McCauley et al., 2021; Rovers et al., 2016; Strkljevic et al., 2024; Umar et al., 2022).

Instrument development proceeded in four stages. First, the research team defined the construct as the perceived multidimensional impact of hospital-based volunteering on the volunteer during armed conflict. Five a priori domains were specified: Social impact, Mental impact, Physical impact, Academic impact, and Wellbeing and fulfillment. Second, candidate items were generated through a focused review of the volunteering, medical education, disaster-response, trauma, burnout, and post-traumatic-growth literature. Third, informal consultations were conducted with Gaza-based medical students and clinicians to identify experiences that might not be adequately represented in the literature developed in stable or post-crisis environments. These consultations highlighted peer solidarity, communication with patients and families during crises, exposure to severe injury and death, physical exhaustion, sleep disruption, interruption of formal education, clinical learning, and willingness to volunteer again. The consultations informed item wording and contextual relevance but were not treated as formal qualitative data.

Fourth, the candidate items were reviewed iteratively by a multidisciplinary research team with expertise in psychiatry, medical education, psychometrics, and conflict health. The review examined conceptual relevance, comprehensibility, contextual appropriateness, duplication, ambiguity, and correspondence between each item and its intended domain. Items were revised or removed through consensus. This was a qualitative expert-review process; a formal item-level or scale-level content-validity index was not calculated. The process resulted in a preliminary 28-item questionnaire that was administered to participants.

The original questionnaire comprised eight Social items, six Mental items, two Physical items, eight Academic items, and four Wellbeing and fulfillment items. The Social domain assessed interpersonal connection, peer support, teamwork, communication, isolation, appreciation of healthcare workers, interpersonal conflict, and community belonging. The Mental domain assessed working under pressure, empowerment, hopelessness, emotional demands, helplessness, and distress arising from exposure to war-related injury or death. The Physical domain assessed exhaustion and sleep disturbance. The Academic domain assessed history-taking, physical examination, emergency diagnosis, procedural skills, concentration, missed academic activities, reintegration into academic life, and career interests. The Wellbeing domain assessed access to psychological support, support from peers and supervisors, willingness to recommend volunteering, and willingness to volunteer again.

All 28 items were presented as first-person statements and rated on a five-point Likert scale ranging from 1 (strongly disagree) to 5 (strongly agree). Eleven negatively worded items—Social5, Social7, Mental3–Mental6, Physical1–Physical2, and Academic5–Academic7—were identified for reverse scoring. Thus, after reverse scoring, higher domain scores represented more favorable perceived outcomes, whereas lower Mental and Physical scores represented greater psychological and physical burden.

The initial 28-item questionnaire was subjected to exploratory factor analysis. Item-retention decisions considered primary factor loadings, cross-loadings, communalities, redundancy, conceptual coherence, and domain coverage. Twelve items—Social5–Social8, Mental1–Mental2, Academic4–Academic8, and Wellbeing1—were removed during refinement. The final 16-item VIS comprised four Social items (Social1–Social4), four Mental items (Mental3–Mental6), two Physical items (Physical1–Physical2), three Academic items (Academic1–Academic3), and three Wellbeing items (Wellbeing2–Wellbeing4). The Mental and Physical items in the final instrument were reverse-scored. Domain scores were calculated as the mean of their constituent items, with higher scores indicating stronger Social, Academic, and Wellbeing benefits and better Mental and Physical outcomes—that is, lower psychological and physical burden. The final five-domain structure was subsequently evaluated through confirmatory analysis. The original structure of the VIS and the items retained or removed during psychometric refinement are summarized in Supplementary Table S1.

### 2.4. Data Collection Procedures

Data were collected using a structured, anonymous online questionnaire administered via Google™ Forms. In addition to the VIS, the survey collected information on sociodemographic characteristics (age, sex, academic level, household size), volunteering characteristics (duration, hospital site, department), and war-related exposures (e.g., displacement, exposure to destruction, loss of colleagues). Participation was voluntary, and respondents provided electronic informed consent prior to accessing the questionnaire. To reduce response bias, no incentives were offered, and participants were assured that their responses would not affect their academic standing or volunteering roles.

### 2.5. Statistical Analysis

Analyses were conducted using IBM SPSS Statistics version 27 and SmartPLS version 4 (Ringle et al., 2024). Participant and volunteering characteristics were summarized using frequencies and percentages for categorical variables. Continuous variables were summarized using means, standard deviations, medians, interquartile ranges, and observed ranges. For multiple-response variables, including clinical departments, hospitals, and war-related experiences, each response option was coded separately as selected or not selected; consequently, the percentages within these categories may exceed 100%.

Subscale means, standard deviations, and score ranges were calculated for the five VIS domains. Negatively worded Mental and Physical items were reverse-scored before calculating their respective subscale means; therefore, higher Mental and Physical scores indicate more favorable outcomes or lower burden. Associations between VIS domains and continuous participant characteristics were examined using Pearson correlations. Group comparisons were conducted using Welch’s independent-samples *t*-test because several comparisons involved unequal group sizes and variances. Cohen’s *d* was reported as the standardized effect size. All tests were two-sided, with *p* < 0.05 considered statistically significant. Because these analyses were exploratory, emphasis was placed on the direction and magnitude of effects in addition to statistical significance.

To evaluate whether these impact domains were empirically distinguishable, exploratory factor analysis (EFA) was conducted using principal axis factoring with Promax rotation. Factorability was assessed using the Kaiser–Meyer–Olkin measure and Bartlett’s test of sphericity. The number of factors retained was guided by eigenvalues, the scree plot, conceptual interpretability, primary loadings of at least 0.40, and minimal cross-loadings. Model fit indices were also examined.

Confirmatory factor analysis using partial least squares structural equation modeling (PLS-SEM) then evaluated the resulting structure under non-normality and the available sample size. Internal consistency was assessed using Cronbach’s alpha, McDonald’s omega, and composite reliability; convergent and discriminant validity were examined using average variance extracted, the Fornell–Larcker criterion, and heterotrait–monotrait ratios.

Item analysis was performed on all 28 initially administered items after applying the specified reverse scoring. For each item, the mean, standard deviation, skewness, excess kurtosis, corrected item–total correlation within its originally hypothesized domain, and Cronbach’s alpha if the item was deleted were calculated. Horn’s parallel analysis was also performed to determine the number of factors supported by the data (Horn, 1965; Hayton et al., 2004). Eigenvalues from the observed 28-item Pearson correlation matrix were compared with the 95th-percentile eigenvalues obtained from 1000 normally distributed random datasets with the same dimensions (*n* = 169 and 28 variables). A factor was retained when its observed eigenvalue exceeded the corresponding random-data eigenvalue. Parallel analysis is generally considered more accurate than reliance solely on the eigenvalue-greater-than-one criterion (Horn, 1965; Hayton et al., 2004).

### 2.6. Ethical Considerations

The study was conducted in accordance with the Declaration of Helsinki. Ethical approval was obtained from the Institutional Review Board of the Islamic University of Gaza (approval letter 2025/3, dated 8 May 2025). All participants received detailed information about the study objectives, procedures, and potential risks before participation. Informed consent was obtained electronically, and data were collected anonymously. Participants were provided with contact information for local mental health support services in the event that survey participation elicited emotional distress.

## 3. Results

### 3.1. Participant and Volunteering Characteristics

A total of 169 medical-student volunteers were included in the analysis. Their mean age was 23.43 years (SD = 1.58; median = 24; IQR = 22–25; range = 20–29), and the mean household size was 6.76 persons (SD = 2.36; median = 7; IQR = 5–8; range = 1–15). Participants had volunteered for a mean of 26.40 weeks (SD = 17.17; median = 24; IQR = 12–40; range = 4–80). Monthly family income was reported by 156 participants and showed considerable variability (M = 2592.18 NIS, SD = 2977.82; median = 2000; IQR = 900–3000; range = 0–18,000).

Most participants were female (68.6%), studying at the clinical level (89.9%), enrolled at the Islamic University of Gaza (67.5%), and single (94.1%). Approximately one-third had an immediate family member working in healthcare (34.3%), while 21.3% had volunteered in a hospital before the war. Emergency or trauma care was the most frequently reported volunteering department (71.6%), followed by surgical departments (39.6%) and non-surgical departments (30.2%). Participants volunteered across numerous hospitals, most commonly Al-Aqsa Hospital (39.6%), Nasser Hospital (17.2%), Al-Shifa Hospital (16.6%), and the European Gaza Hospital (16.6%).

War-related exposure was extensive. Most participants had been displaced from their homes (85.8%), experienced shortages of food, water, or essential supplies (84.6%), lost a medical-school colleague (77.5%), or lost a friend (71.6%). More than one-third reported that their home had been destroyed or severely damaged (35.5%), and 45.6% reported partial damage to their home. Complete participant characteristics are presented in Supplementary Table S2.

### 3.2. Item Analysis and Parallel Analysis

Item-level descriptive statistics for the original 28-item pool are presented in Supplementary Table S3. Item means ranged from 2.19 to 4.54, and standard deviations ranged from 0.56 to 1.10. Skewness ranged from −1.01 to 0.66, while excess kurtosis ranged from −0.96 to 1.96, indicating no substantial univariate distributional abnormalities. Internal-consistency analysis of the originally hypothesized domains produced Cronbach’s alpha coefficients of 0.661 for Social impact, 0.579 for Mental burden, 0.643 for Physical burden, 0.503 for Academic development, and 0.649 for Wellbeing and fulfillment. Several subsequently removed items demonstrated weak or negative corrected item–total correlations. In particular, Mental1 (*r* = −0.061), Mental2 (*r* = 0.050), and Academic8 (*r* = −0.100) showed particularly poor consistency with their proposed domains. Removing Mental1 increased the Mental-domain alpha from 0.579 to 0.644, removing Mental2 increased it to 0.619, and removing Academic8 increased the Academic-domain alpha from 0.503 to 0.600. Removal of Wellbeing1 increased the Wellbeing-domain alpha from 0.649 to 0.702. Social5 and Social7 also demonstrated comparatively weak corrected item–total correlations of 0.245 and 0.253, respectively. These findings, considered together with factor loadings, cross-loadings, redundancy, and conceptual relevance, informed the item-reduction process.

**Figure 1:**
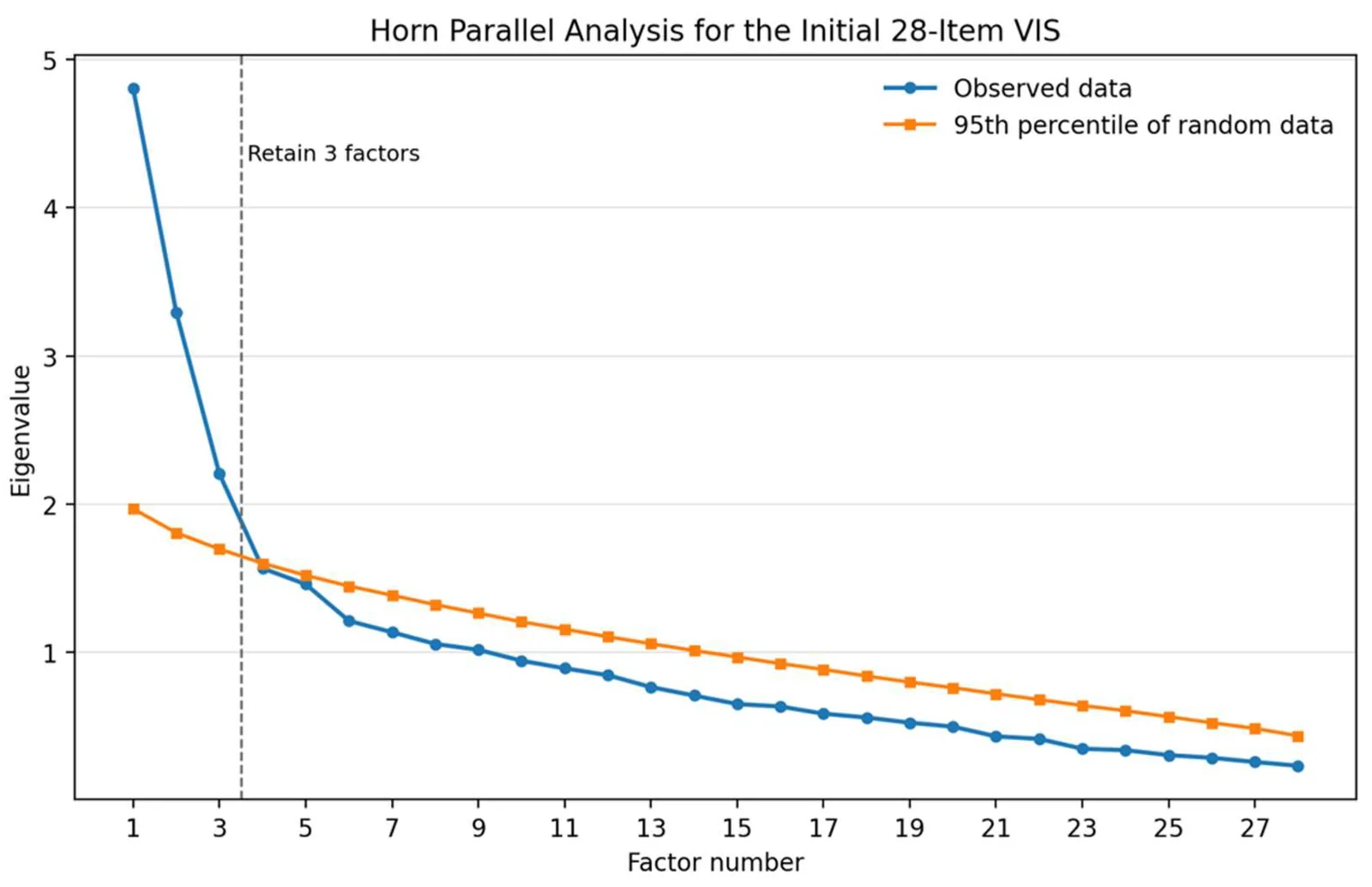
Horn parallel analysis of the initial 28-item Volunteering Impact Scale. Observed eigenvalues were compared with the 95th-percentile eigenvalues obtained from 1000 random datasets containing 169 observations and 28 variables. The first three observed eigenvalues exceeded the corresponding random eigenvalues, supporting retention of three factors.

Horn’s parallel analysis recommended retaining three factors from the initial 28-item pool (Figure 1). The first three observed eigenvalues—4.804, 3.292, and 2.204—exceeded the corresponding 95th-percentile random eigenvalues of 1.968, 1.807, and 1.697. However, the fourth observed eigenvalue of 1.567 was lower than the corresponding random eigenvalue of 1.601, and subsequent observed eigenvalues also failed to exceed their random-data counterparts. Thus, parallel analysis provided empirical support for three factors rather than the five factors suggested by the eigenvalue-greater-than-one criterion. Nevertheless, because the instrument was constructed around five prespecified conceptual domains, the five-domain solution was retained for subsequent theory-driven evaluation.

### 3.3. Factor Structure: Exploratory Factor Analysis

The initial 28-item scale was administered to participants. Item reduction was undertaken iteratively using a combination of statistical and conceptual criteria, including weak primary loadings, problematic cross-loadings, low communalities, redundancy, and limited conceptual contribution. This process resulted in the removal of 12 items. The retained 16 items formed the five-domain solution subsequently evaluated through confirmatory factor analysis.

An exploratory factor analysis (EFA) using principal axis factoring with Promax rotation was conducted to examine the underlying structure of the VIS (*n* = 169). Factorability of the initial 28-item correlation matrix was supported by a Kaiser–Meyer–Olkin (KMO) measure of 0.652, indicating modest but acceptable sampling adequacy, and a significant Bartlett’s test of sphericity, χ^2^(378) = 2194.96, *p* < 0.001. Mardia’s test indicated significant multivariate skewness and kurtosis (skewness = 187.69, *p* < 0.001; kurtosis = 9.07, *p* < 0.001), demonstrating deviation from multivariate normality.

Five factors with eigenvalues greater than 1 were retained, explaining 51.2% of the total variance after rotation. The final 16-item solution comprised five interpretable domains: Social impact (four items), Mental burden (four items), Physical burden (two items), Academic development (three items), and Wellbeing and fulfillment (three items).

Within the final solution, absolute primary loadings ranged from 0.23 to 0.89, and communalities ranged from 0.24 to 0.72 (Table 1). Most retained items met the prespecified loading criterion of 0.40. However, three Social items had weaker primary loadings and were retained provisionally because they represented conceptually important aspects of social connectedness, teamwork, and communication that were not adequately captured by the remaining domains. Cross-loadings were generally limited, and inter-factor correlations ranged from 0.13 to 0.35, suggesting that the five domains were related but distinguishable. Fit indices for the exploratory solution were mixed: SRMR indicated acceptable residual fit (0.060), whereas RMSEA = 0.114 (90% CI [0.106, 0.124]), TLI = 0.529, and CFI = 0.699 indicated inadequate overall fit. Accordingly, the exploratory structure was treated as preliminary and evaluated further through confirmatory analysis.

### 3.4. Confirmatory Factor Analysis

**Figure 2:**
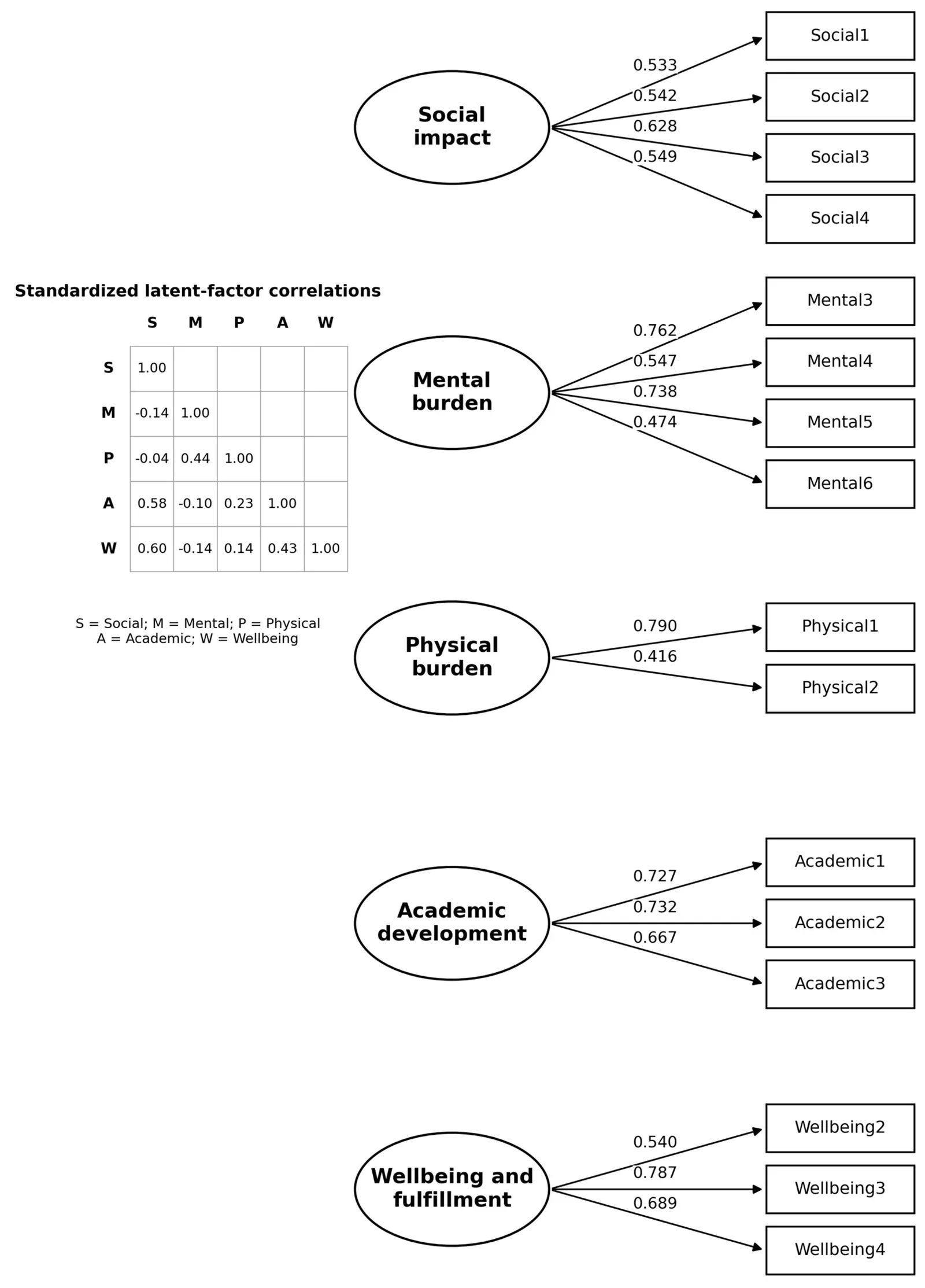
Proposed five-factor measurement model of the 16-item Volunteering Impact Scale. Ellipses represent latent domains, rectangles represent observed items, single-headed arrows display standardized factor loadings. The lower-triangular inset displays standardized correlations among the latent domains. Item residuals are omitted for visual clarity. Mental3–Mental6 and Physical1–Physical2 are reverse-scored for subscale calculation, so higher Mental and Physical scores indicate lower burden. Domain labels describe the content assessed, not the direction of scoring.

Confirmatory factor analysis was conducted to evaluate the proposed five-domain structure of the VIS. The model was derived from the original 28-item questionnaire following the removal of 12 items during exploratory item reduction. The resulting 16-item model specified five correlated first-order latent factors: Social impact, measured by Social1–Social4; Mental burden, measured by Mental3–Mental6; Physical burden, measured by Physical1–Physical2; Academic development, measured by Academic1–Academic3; and Wellbeing and fulfillment, measured by Wellbeing2–Wellbeing4. Each observed item was specified to load on only its corresponding latent factor, cross-loadings were fixed to zero, and correlations among the five latent factors were freely estimated. The revised black-and-white measurement model is presented in Figure 2.

The five-factor model demonstrated acceptable overall fit: χ^2^ = 150.40, *df* = 95, χ^2^/*df* = 1.58, *p* < 0.001; RMSEA = 0.059, 90% CI [0.040, 0.076]; SRMR = 0.075; GFI = 0.896; AGFI = 0.852; TLI = 0.888; and CFI = 0.911. Standardized factor loadings ranged from 0.41 to 0.79. Internal-consistency estimates were modest to acceptable, with Cronbach’s alpha coefficients ranging from 0.643 to 0.752, McDonald’s omega coefficients from 0.644 to 0.754, and composite reliability estimates from 0.644 to 0.754. Standardized correlations among the latent factors ranged from −0.14 to 0.60; the strongest were Social impact with Wellbeing and fulfillment (*r* = 0.60), Social impact with Academic development (*r* = 0.58), and Mental burden with Physical burden (*r* = 0.44).

Evidence of convergent validity was mixed. The Academic factor met the conventional AVE criterion of 0.50 (AVE = 0.507), whereas the Social (AVE = 0.332), Mental (AVE = 0.409), Physical (AVE = 0.475), and Wellbeing (AVE = 0.460) factors fell below this threshold. Composite reliability exceeded 0.70 for the Mental, Academic, and Wellbeing factors but was lower for the Social (CR = 0.666) and Physical (CR = 0.644) factors. Accordingly, convergent validity was strongest for Academic development, broadly acceptable but incomplete for Mental burden and Wellbeing, and comparatively weak for Social impact and Physical burden.

Discriminant validity was supported more consistently. Under the Fornell–Larcker criterion, the square root of each construct’s AVE was greater than its correlations with the other constructs. In addition, all HTMT ratios were below the conservative threshold of 0.85, ranging from 0.18 to 0.70. These findings indicate that the five domains were empirically distinguishable, despite the limitations observed in the convergent validity of some brief subscales.

Overall, the CFA fit indices and discriminant-validity findings provided preliminary support for distinguishing the five theoretically proposed domains. However, the mixed convergent-validity results, modest reliability of the Social and Physical subscales, and discrepancy between the theoretically specified model and the factor-retention results indicate that the five-domain structure should be considered provisional. Further refinement and independent validation of the instrument are required.

### 3.5. Descriptive Statistics and Score Interpretation

Table 2 summarizes the distributions of the five VIS subscale scores alongside the reliability and convergent-validity estimates discussed in Section 3.4.

Mean scores were highest for Social (M = 4.31, SD = 0.45) and Academic (M = 4.19, SD = 0.56), followed by Wellbeing (M = 3.96, SD = 0.58). Mean reverse-scored Mental and Physical scores were 2.71 (SD = 0.74) and 2.60 (SD = 0.86), respectively. Observed scores ranged from 2.50 to 5.00 for Social, 1.00 to 4.50 for Mental, 1.00 to 5.00 for Physical, 2.33 to 5.00 for Academic, and 2.00 to 5.00 for Wellbeing. Only the Physical subscale covered the full theoretical response range of 1–5. Higher Social, Academic, and Wellbeing scores indicate greater perceived benefits of volunteering, whereas higher reverse-scored Mental and Physical scores indicate lower reported psychological and physical burden.

### 3.6. Differences by Participant Characteristics

Exploratory analyses identified several small-to-moderate associations between participant characteristics and VIS scores. Female participants had lower Mental scores than male participants (M = 2.63, SD = 0.76 versus M = 2.87, SD = 0.69), Welch’s *t*(109.61) = −2.00, *p* = 0.048, *d* = −0.32. Because the Mental items were reverse-scored, this indicates greater psychological burden among female participants. Female participants also had slightly lower Academic scores than male participants (M = 4.14, SD = 0.58 versus M = 4.31, SD = 0.52), Welch’s *t*(111.72) = −1.99, *p* = 0.049, *d* = −0.32.

Participants with an immediate family member working in healthcare had lower Physical scores than those without a healthcare-worker family member (M = 2.31, SD = 0.84 versus M = 2.74, SD = 0.84), Welch’s *t*(115.06) = −3.18, *p* = 0.002, *d* = −0.52, indicating greater physical burden. Participants who volunteered in surgical departments reported higher Wellbeing scores than those who did not (M = 4.12, SD = 0.64 versus M = 3.86, SD = 0.52), Welch’s *t*(121.74) = 2.88, *p* = 0.005, *d* = 0.47.

Longer volunteering duration was associated with lower Physical scores (*r* = −0.30, *p* < 0.001), but higher Academic (*r* = 0.21, *p* = 0.005) and Wellbeing scores (*r* = 0.29, *p* < 0.001). Thus, longer participation was associated with greater physical burden alongside stronger perceived academic and wellbeing benefits. Age (*r* = 0.17, *p* = 0.027) and household size (*r* = 0.17, *p* = 0.031) were weakly associated with higher Academic scores.

Participants who reported losing a medical-school colleague had higher Social scores than those who did not (M = 4.35, SD = 0.44 versus M = 4.16, SD = 0.48), Welch’s *t*(55.87) = 2.09, *p* = 0.041, *d* = 0.41. Differences were also observed in Physical scores according to hospital and destruction exposure. Participants who volunteered at the Indonesian Hospital had higher Physical scores than other participants (M = 3.19, SD = 0.85 versus M = 2.54, SD = 0.85), Welch’s *t*(14.03) = 2.63, *p* = 0.020, *d* = 0.77. Participants whose homes had been destroyed or severely damaged also had higher Physical scores than those without this exposure (M = 2.82, SD = 1.04 versus M = 2.47, SD = 0.72), Welch’s *t*(90.67) = 2.28, *p* = 0.025, *d* = 0.41. Because higher Physical scores indicate lower burden, these latter findings should not be interpreted as evidence that these exposures caused greater physical strain. All associations were exploratory and are summarized in Table 3.

## 4. Discussion

This study presents an initial multidimensional evaluation of healthcare volunteering in an active war setting and introduces a preliminary framework for assessing its perceived benefits and burdens. The findings suggest that positive and negative experiences can coexist rather than representing mutually exclusive outcomes. This coexistence is also recognized in Stamm’s Professional Quality of Life (ProQOL) framework, which assesses compassion satisfaction, burnout, and secondary traumatic stress as distinct dimensions. High compassion satisfaction can coexist with high secondary traumatic stress, indicating that fulfillment from helping does not necessarily imply an absence of distress (Stamm, 2010). The VIS findings are consistent with this perspective, although its domains are not equivalent to the ProQOL subscales. This pattern also aligns with trauma scholarship emphasizing heterogeneous responses to adversity shaped by role, meaning, and context (Bonanno, 2004; Thormar et al., 2010; Weiss & Berger, 2010). For example, longer volunteering duration was associated with greater physical burden (reverse-scored Physical domain: *r* = −0.30), but also with greater perceived academic (*r* = 0.21) and wellbeing-related benefits (*r* = 0.29). Conversely, participants whose homes had been destroyed or severely damaged reported lower physical burden than those without this exposure (reverse-scored Physical means: 2.82 versus 2.47). These contrasting associations illustrate the complexity of volunteers’ experiences; they do not establish that prolonged volunteering caused these outcomes or that destruction exposure was protective.

The coexistence of perceived benefits and burdens may reflect the different demands and opportunities associated with clinical helping roles. Opportunities for learning, social connection, and professional development may occur alongside emotional demands, disrupted sleep, and physical strain (Bazan et al., 2021; Dell et al., 2014; Lu et al., 2018; Thormar et al., 2010; Umar et al., 2022). Assessing these dimensions separately may therefore provide a more informative account of volunteering experiences than a single overall impact score.

The detailed sample distribution provides important context for interpreting these findings. The sample was predominantly female, clinically advanced, and enrolled at the Islamic University of Gaza. This composition likely reflects both the structure of the available volunteer workforce and the recruitment pathways used during the conflict. Clinical-level students may have had greater opportunities to undertake direct patient-care tasks than preclinical students, potentially contributing to the high Academic scores (Bazan et al., 2021; Lu et al., 2018; Rovers et al., 2016). However, the limited representation of preclinical students and students from the second medical school restricts the extent to which the findings can be generalized across all medical students in Gaza.

Participants with an immediate family member working in healthcare reported lower reverse-scored Physical scores than those without such a connection, indicating greater physical burden (M = 2.31 versus 2.74). Family connections to healthcare may influence not only access to volunteering but also the responsibilities and expectations surrounding participation. For example, greater familiarity with clinical environments or connections to healthcare personnel could facilitate involvement in more demanding tasks, while perceived family or professional expectations might encourage sustained participation despite fatigue. Volunteers may also combine their clinical responsibilities with additional family demands when relatives are themselves working under emergency conditions. Alternatively, healthcare-worker relatives could provide practical advice, emotional support, and assistance in navigating clinical roles. Thus, family healthcare ties may offer resources while also creating additional demands; the observed association does not establish that these ties are inherently disadvantageous.

These explanations remain tentative. The study did not measure shift length, workload, task allocation, family expectations, caregiving responsibilities, or the type and availability of support provided by healthcare-worker relatives. The association may therefore reflect unmeasured differences in volunteering conditions rather than an independent effect of family connections. Future studies should examine these mechanisms directly and assess whether the association persists after accounting for clinical responsibilities, volunteering intensity, and war-related exposures. Separately, volunteering in surgical departments was associated with higher Wellbeing scores, possibly reflecting opportunities to contribute, acquire procedural experience, and observe the immediate effects of care (Bazan et al., 2021; Lu et al., 2018; McCauley et al., 2021); this interpretation likewise requires further investigation.

Unexpectedly, participants whose homes had been destroyed or severely damaged and those who volunteered at the Indonesian Hospital in Beit Lahia, northern Gaza, had higher reverse-scored Physical scores, indicating lower—not higher—reported physical burden. These findings may reflect selection effects, adaptation, differences in role allocation, or unmeasured organizational support and workload conditions. Similar research has shown that the effects of disaster volunteering vary according to exposure severity, preparedness, role clarity, and organizational support (Aldamman et al., 2019; Thormar et al., 2010). The present findings should not be interpreted as evidence that destruction exposure or working in a particular hospital was physically protective. Given the unequal subgroup sizes and cross-sectional design, these associations require replication in larger samples.

Female participants reported slightly lower Mental and Academic scores than male participants, although both effects were small. Because the Mental items were reverse-scored, the lower mean among women indicates greater reported psychological burden. This pattern is consistent with broader evidence of gendered differences in trauma responses, coping strategies, and professional socialization (Dell et al., 2014; Tamres et al., 2002; Umar et al., 2022). Women in healthcare and humanitarian roles may report greater emotional distress because of differences in caregiving demands, empathic engagement, social expectations, and exposure patterns (Aldabbour et al., 2024; Tamres et al., 2002). Nevertheless, these findings should not be interpreted as evidence of inherent sex-based vulnerability. Differences in assigned duties, exposure intensity, caregiving responsibilities, supervision, or access to clinical learning opportunities may also have contributed (Bazan et al., 2021; Lu et al., 2018; Rovers et al., 2016). The results support the importance of sex-sensitive volunteer-support structures while cautioning against one-size-fits-all assumptions (Aldamman et al., 2019; Dell et al., 2014).

Volunteering duration was associated with lower Physical scores but higher Academic and Wellbeing scores. These findings indicate that sustained participation may simultaneously increase physical burden and strengthen clinical learning, perceived contribution, and personal fulfillment. This concurrent pattern supports the central rationale of the VIS: positive and negative consequences are not mutually exclusive. Similar patterns have been reported among healthcare workers and humanitarian responders, for whom cumulative exposure may increase exhaustion while also strengthening purpose, professional identity, and perceived contribution (McCauley et al., 2021; Shultz & Forbes, 2014; Thormar et al., 2010). Participants may therefore gain competence and meaning while becoming increasingly fatigued through prolonged shifts, disrupted sleep, resource scarcity, and continuing exposure to suffering (Aldamman et al., 2019; Dell et al., 2014; Thormar et al., 2010).

Participants who had lost a medical-school colleague reported higher Social scores than those who had not experienced such a loss. This association may reflect increased solidarity, peer identification, collective coping, or meaning-making following shared bereavement (Staub & Vollhardt, 2008; Thoits, 2012; Weiss & Berger, 2010). However, the cross-sectional design cannot establish whether shared loss strengthened social connectedness or whether students with stronger peer connections perceived and reported their experiences differently. This finding should therefore be interpreted cautiously and examined further in longitudinal research.

### 4.1. Contribution to the Volunteering and Humanitarian Literature

To clarify the contribution of the VIS, it is important to situate its intended measurement purpose in relation to established volunteering instruments. The VIS should be situated alongside existing volunteering questionnaires according to their intended measurement purposes. The Volunteer Functions Inventory assesses the motivational functions served by volunteering, including social, career, understanding, protective, enhancement, and values-related functions (Clary et al., 1998). Its social and career-related content overlaps conceptually with the VIS Social and Academic domains, but the instruments address different questions: motivations for volunteering versus perceived consequences of participation. The Volunteer Satisfaction Index assesses organizational support, participation efficacy, empowerment, and group integration (Galindo-Kuhn & Guzley, 2001). Its emphasis on support and integration overlaps with elements of the VIS Social and Wellbeing domains. These similarities indicate that the VIS does not introduce entirely new constructs; rather, it brings selected constructs together within a context-specific assessment of hospital-based volunteering during armed conflict.

The International Volunteer Impacts Survey provides a closer comparison because it explicitly assesses volunteering outcomes, including social, civic, intercultural, and developmental consequences (Lough et al., 2009; McBride et al., 2012). Accordingly, the potential contribution of the VIS is not the assessment of volunteering impact itself, but its brief combination of perceived clinical learning and social benefits with psychological and physical burden among local medical-student volunteers. Whether this combination provides incremental value beyond established measures remains untested. Direct comparisons are required to determine whether the VIS captures additional information, offers practical advantages, or reproduces constructs already assessed adequately by other instruments.

Item reduction also narrowed the content represented by several VIS domains. The retained Academic items concern perceived improvements in history-taking, physical examination, and emergency diagnosis; they therefore represent perceived clinical-skill development more directly than academic development in its broader sense. Similarly, the retained Wellbeing items concern perceived support, willingness to recommend volunteering, and willingness to volunteer again. These items may capture support and favorable appraisal of volunteering more directly than general psychological wellbeing. The two Physical items assess exhaustion and sleep disturbance rather than the full range of possible physical consequences. These distinctions are important when interpreting subscale names and support further content-validation work before broader claims about domain coverage are made.

### 4.2. Psychometric Evidence and Priorities for Further Validation

The initial psychometric evaluation provided encouraging evidence for the VIS while identifying specific priorities for refinement. The five-domain model offered a theoretically interpretable representation of volunteering experiences, and the confirmatory analysis yielded several favorable fit indices. Nevertheless, parallel analysis of the original 28-item pool favored three factors, indicating that the distinction between broader empirical dimensions and the five proposed domains warrants further examination. The exploratory model also showed inadequate overall fit, and three Social items had primary loadings of approximately 0.23–0.25. These items were retained provisionally to preserve coverage of social connectedness, teamwork, and communication. Independent studies comparing alternative structures and using factor-retention procedures appropriate for ordinal responses would help clarify whether these domains are best represented separately or within broader dimensions.

Reliability and convergent-validity findings suggest priorities for targeted refinement. Academic development showed the strongest convergent-validity evidence, whereas Social impact and Physical burden warrant particular attention to measurement precision and item coherence. The more favorable reliability findings for Mental and Wellbeing were accompanied by incomplete evidence of convergence, underscoring the need to consider these properties together. Reviewing item wording and evaluating additional indicators could strengthen the weaker domains while preserving the instrument’s brevity and contextual relevance.

The reported Fornell–Larcker and HTMT results provided complementary evidence that the proposed domains capture distinguishable aspects of volunteering. This evidence supports their continued investigation, although it does not establish adequate convergent validity for every domain or settle the optimal factor structure. Because exploratory and confirmatory analyses used the same sample, the findings constitute an initial within-sample evaluation rather than independent confirmation. Replication, comparison with established instruments, and assessment of temporal stability are therefore the next steps toward determining the VIS’s suitability for broader research and practice applications.

### 4.3. Implications

The findings have several implications for research, practice, and policy. For researchers, the VIS enables a systematic investigation of the impact of volunteering without privileging benefits over burdens, facilitating more balanced theory development and more precise modeling of volunteer experiences in high-adversity settings (Rovers et al., 2016; Strkljevic et al., 2024). For humanitarian organizations and academic institutions, the results underscore the need to integrate monitoring, rest periods, and psychosocial support into volunteer programs rather than assuming that volunteers possess intrinsic resilience (Dell et al., 2014; Thormar et al., 2010). At a policy level, the study supports calls for ethically informed volunteer deployment frameworks that recognize both the contributions and costs borne by volunteers in conflict settings (Bauer, 2017; Irfan et al., 2025; Rovers et al., 2016). The VIS may also warrant adaptation for non-war, high-stress volunteering environments, including volunteer firefighting and paramedic services. Such adaptation would require reviewing the war-specific wording of Mental6 and replacing medical-student-specific Academic items with relevant role-related learning or skill-development items. Consultation with the intended volunteer groups, cognitive interviewing, and independent psychometric evaluation would be needed before assuming that the revised instrument measures comparable domains across settings.

### 4.4. Limitations

Several limitations should be considered when interpreting these findings. Although the study captures substantial contextual variation, unmeasured factors such as personality traits, coping styles, and informal support networks may have influenced responses (Shultz & Forbes, 2014; Tamres et al., 2002). In addition, although the VIS was developed in a war-affected setting, further validation is required to assess its performance among other healthcare students and across different humanitarian contexts and volunteer populations. Content review was conducted qualitatively through multidisciplinary consensus, without calculation of a formal content-validity index or cognitive interviewing; future validation should therefore include structured expert ratings and cognitive interviews with healthcare-student volunteers. The study did not administer established external instruments measuring related constructs. Consequently, criterion-related validity and convergent validity based on associations with independent measures could not be evaluated. The AVE, composite reliability, Fornell–Larcker, and HTMT analyses assess the internal measurement properties of the VIS but do not establish its relationship with external constructs. Future studies should therefore examine correlations with validated measures of psychological distress, traumatic stress, burnout, physical fatigue, social support, wellbeing, professional development, and academic self-efficacy. Testing associations with theoretically unrelated measures would also provide evidence of external discriminant validity. Predictive and known-groups validity should be examined in independent samples and, where possible, across different humanitarian and volunteering contexts. The absence of open-ended survey questions limited opportunities for participants to describe experiences not captured by the predefined items. Future research should incorporate qualitative interviews with volunteers representing different clinical roles, volunteering durations, and levels of reported benefit and burden.

## 5. Conclusions

Volunteering in war-affected contexts represents a complex engagement characterized by concurrent benefits and burdens rather than a unidimensional outcome. The VIS offers a preliminary, context-sensitive framework for assessing perceived benefits and burdens of hospital-based volunteering among medical students in a war-affected setting. However, uncertainty about factor structure, limited convergent validity, modest reliability in some domains, and the absence of independent and external validation restrict its current interpretation. Further item refinement and validation are required before routine assessment or individual-level decision-making can be recommended. Its potential contribution lies in complementing established measures through a brief assessment of volunteering-related experiences specific to this context.

## Figures and Tables

**Table 1:** Rotated Pattern Matrix (Principal Axis Factoring, Promax; *n* = 169). Columns show loadings per domain.

Item	Social	Mental	Physical	Academic	Wellbeing	Primary Loading	Max Cross Loading
Social1—Volunteering enhanced my ability to connect with others.	0.234	0.004	0.039	−0.144	−0.190	0.234	0.190
Social2—Volunteering increased my appreciation for peer support.	0.487	−0.023	−0.020	−0.073	−0.068	0.487	0.073
Social3—Volunteering improved my ability to work collaboratively in a team.	0.248	0.181	0.087	−0.146	−0.192	0.248	0.192
Social4—Volunteering strengthened my communication skills with patients and families during crisis situations.	0.234	0.059	0.019	−0.150	−0.181	0.234	0.181
Mental3—Volunteering exposed me to situations that made me feel hopeless.	0.080	0.766	−0.016	0.028	−0.162	0.766	0.162
Mental4—Volunteering was emotionally demanding.	−0.175	0.483	−0.178	0.024	0.022	0.483	0.178
Mental5—Volunteering exposed me to situations that made me feel helpless.	−0.029	0.827	−0.036	−0.084	0.064	0.827	0.084
Mental6—Volunteering caused me psychological distress due to exposure to war-related injuries or death.	−0.093	0.433	−0.303	0.057	0.009	0.433	0.303
Physical1—I felt physically exhausted after volunteering shifts.	0.124	0.321	−0.515	0.082	0.131	0.515	0.321
Physical2—I experienced sleep disturbances during the volunteering period.	−0.020	0.020	−0.893	0.035	−0.005	0.893	0.035
Academic1—Volunteering improved my ability to take a patient’s history.	0.091	0.007	0.133	−0.494	−0.051	0.494	0.133
Academic2—Volunteering improved my physical examination skills.	−0.041	0.147	0.146	−0.501	−0.130	0.501	0.147
Academic3—Volunteering improved my ability to diagnose common emergency conditions.	0.180	−0.005	−0.024	−0.414	−0.130	0.414	0.180
Wellbeing2—Volunteering made me feel supported by my peers and supervisors.	−0.085	0.209	0.100	−0.052	−0.402	0.402	0.209
Wellbeing3—Volunteering encouraged me to recommend similar experiences to others.	0.094	0.015	0.011	−0.143	−0.496	0.496	0.143
Wellbeing4—Volunteering motivated me to participate again in similar future situations.	0.070	0.021	0.012	−0.077	−0.534	0.534	0.077

Notes: Complete cases (*n* = 169). PrimaryFactor = factor with largest absolute loading. MaxCrossLoading = second-largest absolute loading. EFA diagnostics: KMO = 0.652; Bartlett’s χ^2^(378) = 2194.96, *p* < 0.001; five factors retained; rotated variance explained = 51.2%; RMSEA = 0.114 [0.106, 0.124]; SRMR = 0.060; TLI = 0.529; CFI = 0.699.

**Table 2:** Descriptive statistics, internal consistency, and convergent validity indices for the VIS five subscales.

Sub-Scale	*n*	Mean	Median	SD	Min	Max	α	ω	CR	AVE
Social	169	4.305	4.250	0.451	2.500	5.000	0.663	0.665	0.666	0.332
Mental	169	2.706	2.750	0.744	1.000	4.500	0.720	0.726	0.726	0.409
Physical	169	2.595	2.500	0.861	1.000	5.000	0.643	0.644	0.644	0.475
Academic	169	4.193	4.000	0.563	2.333	5.000	0.752	0.754	0.754	0.507
Wellbeing	169	3.963	4.000	0.582	2.000	5.000	0.702	0.714	0.714	0.460

Scores are item means (1–5). α = Cronbach’s alpha; ω = McDonald’s omega; CR = composite reliability; AVE = average variance extracted.

**Table 3:** Significant exploratory associations with VIS domain scores.

Predictor	Outcome	Group Statistics or Association	Test	*p*	Effect
Sex	Mental	Female: 2.63 ± 0.76; male: 2.87 ± 0.69	*t*(109.61) = −2.00	0.048	*d* = −0.32
Sex	Academic	Female: 4.14 ± 0.58; male: 4.31 ± 0.52	*t*(111.72) = −1.99	0.049	*d* = −0.32
Family healthcare worker	Physical	Yes: 2.31 ± 0.84; no: 2.74 ± 0.84	*t*(115.06) = −3.18	0.002	*d* = −0.52
Surgical department	Wellbeing	Yes: 4.12 ± 0.64; no: 3.86 ± 0.52	*t*(121.74) = 2.88	0.005	*d* = 0.47
Indonesian Hospital	Physical	Yes: 3.19 ± 0.85; no: 2.54 ± 0.85	*t*(14.03) = 2.63	0.020	*d* = 0.77
Home destroyed/severely damaged	Physical	Yes: 2.82 ± 1.04; no: 2.47 ± 0.72	*t*(90.67) = 2.28	0.025	*d* = 0.41
Lost medical-school colleague	Social	Yes: 4.35 ± 0.44; no: 4.16 ± 0.48	*t*(55.87) = 2.09	0.041	*d* = 0.41
Volunteering duration	Physical	—	*r* = −0.30	<0.001	Small–moderate
Volunteering duration	Academic	—	*r* = 0.21	0.005	Small
Volunteering duration	Wellbeing	—	*r* = 0.29	<0.001	Small–moderate
Age	Academic	—	*r* = 0.17	0.027	Small
Household size	Academic	—	*r* = 0.17	0.031	Small

Note. Group values are means ± standard deviations. Welch’s independent-samples *t*-tests were used for categorical comparisons. Pearson correlations were used for continuous predictors. Higher Mental and Physical scores indicate better outcomes or lower burden.

## Data Availability

Available from the corresponding author upon reasonable request.

## Supplementary Materials

The following supporting information can be downloaded at: https://www.mdpi.com/article/10.3390/ejihpe16090144/s1, Table S1: Original structure and disposition of the 28 VIS items; Table S2: Demographic, volunteering, and war-exposure characteristics of participants (*n* = 169); Table S3: Item-level descriptive statistics and item analysis for the original 28-item Volunteering Impact Scale pool (*n* = 169).
