# Supplementary material for: A Multidimensional Assessment of the Impact of Healthcare Volunteering in a War-Affected Setting
Source: Eur J Investig Health Psychol Educ. 2026 Sep 19;16(9):144. doi: 10.3390/ejihpe16090144 (PMC13605970; doi:10.3390/ejihpe16090144)
Supplement: Supplementary file 1 [file ejihpe-16-00144-s001.zip › ejihpe-4490391-supplementary.pdf]

Supplementary Table S1. Original structure and disposition of the 28 VIS items.

Original domain	Original items	Number administered	Items retained	Items removed
Social	Social1–Social8	8	Social1–Social4	Social5–Social8
Mental	Mental1–Mental6	6	Mental3–Mental6	Mental1–Mental2
Physical	Physical1–Physical2	2	Physical1–Physical2	None
Academic	Academic1–Academic8	8	Academic1–Academic3	Academic4–Academic8
Wellbeing	Wellbeing1–Wellbeing4	4	Wellbeing2–Wellbeing4	Wellbeing1
Total		28	16	12

Supplementary Table S2. Demographic, volunteering, and war-exposure characteristics of participants (*n* = 169).

Characteristic	Category or statistic	Value
Age, years	Mean ± SD	23.43 ± 1.58
	Median (IQR)	24 (22–25)
	Range	20–29
Sex	Female	116 (68.6%)
	Male	53 (31.4%)
Academic level	Clinical	152 (89.9%)
	Basic sciences/preclinical	17 (10.1%)
University	Islamic University of Gaza	114 (67.5%)
	Al-Azhar University–Gaza	55 (32.5%)
Marital status	Single	159 (94.1%)
	Engaged	5 (3.0%)
	Married without children	3 (1.8%)
	Married with children	2 (1.2%)
Household size	Mean ± SD	6.76 ± 2.36
	Median (IQR)	7 (5–8)
	Range	1–15
Monthly family income, NIS	Valid responses	156
	Mean ± SD	2,592.18 ± 2,977.82
	Median (IQR)	2,000 (900–3,000)
	Range	0–18,000

Characteristic	Category or statistic	Value
Volunteering duration, weeks	Mean ± SD	26.40 ± 17.17
	Median (IQR)	24 (12–40)
	Range	4–80
Immediate family member is a healthcare worker	Yes	58 (34.3%)
	No	111 (65.7%)
Hospital volunteering before the war	Yes	36 (21.3%)
	No	133 (78.7%)
Current location	Gaza	64 (37.9%)
	Middle Gaza	52 (30.8%)
	Khan Younis	29 (17.2%)
	North Gaza	19 (11.2%)
	Outside Gaza	5 (3.0%)
Current living arrangement	Partially damaged home	49 (29.0%)
	Original undamaged home	36 (21.3%)
	With relatives or friends	33 (19.5%)
	Rented home	26 (15.4%)
	Tent or temporary shelter	25 (14.8%)
Volunteering department[1]	Emergency/trauma	121 (71.6%)
	Surgical	67 (39.6%)
	Non-surgical	51 (30.2%)

Characteristic	Category or statistic	Value
	Other	13 (7.7%)
	Intensive care	8 (4.7%)
Hospital[1]	Al-Aqsa Hospital	67 (39.6%)
	Nasser Hospital	29 (17.2%)
	Al-Shifa Hospital	28 (16.6%)
	European Gaza Hospital	28 (16.6%)
	Al-Ahli Arab Hospital	21 (12.4%)
	Indonesian Hospital	13 (7.7%)
	Al-Najjar Hospital	11 (6.5%)
	Other hospitals or medical points	39 (23.1%)
War-related experience[1]	Displaced from home	145 (85.8%)
	Lack of food, water, or supplies	143 (84.6%)
	Lost a medical-school colleague	131 (77.5%)
	Lost a friend	121 (71.6%)
	Lost a second- or third-degree relative	104 (61.5%)
	Home partially damaged	77 (45.6%)
	Home destroyed or severely damaged	60 (35.5%)
	Lost a first-degree relative	25 (14.8%)
	Physically injured	17 (10.1%)
	Detained	6 (3.6%)

Note. Values are presented as *n* (%) unless otherwise stated. IQR = interquartile range; NIS = New Israeli shekel; SD = standard deviation. [1]Multiple responses were permitted; therefore, percentages may exceed 100%. Family-income statistics are based on 156 participants because 13 values were missing.

Supplementary Table S3. Item-level descriptive statistics and item analysis for the original 28-item Volunteering Impact Scale pool (*n* = 169).

Item	Reverse scored	Mean	SD	Observed range	Skewness	Excess kurtosis	Corrected item–total r	α if item deleted
Social1	No	4.54	0.56	3–5	-0.678	-0.602	0.435	0.618
Social2	No	4.07	0.66	2–5	-0.454	0.653	0.348	0.632
Social3	No	4.23	0.68	2–5	-0.668	0.665	0.462	0.604
Social4	No	4.38	0.65	3–5	-0.576	-0.646	0.401	0.620
Social5	Yes	3.82	0.81	2–5	-0.677	0.247	0.245	0.660
Social6	No	3.98	0.81	1–5	-0.775	0.820	0.411	0.615
Social7	Yes	3.36	0.93	1–5	-0.178	-0.630	0.253	0.666
Social8	No	4.10	0.70	1–5	-0.675	1.615	0.362	0.628
Mental1	No	4.34	0.62	3–5	-0.361	-0.649	-0.061	0.644
Mental2	No	3.97	0.67	2–5	-0.331	0.323	0.050	0.619
Mental3	Yes	2.82	1.03	1–5	0.062	-0.509	0.582	0.390
Mental4	Yes	2.58	0.92	1–5	0.324	-0.102	0.418	0.487
Mental5	Yes	2.91	1.10	1–5	-0.138	-0.958	0.529	0.415
Mental6	Yes	2.51	0.99	1–5	0.202	-0.566	0.316	0.535
Physical1	Yes	2.19	0.94	1–5	0.662	0.042	0.477	—
Physical2	Yes	3.00	1.06	1–5	-0.180	-0.865	0.477	—
Academic1	No	4.20	0.69	2–5	-0.825	1.341	0.379	0.422
Academic2	No	4.20	0.70	2–5	-0.408	-0.524	0.293	0.450
Academic3	No	4.18	0.67	2–5	-0.351	-0.278	0.399	0.417
Academic4	No	4.05	0.82	2–5	-0.543	-0.277	0.193	0.483
Academic5	Yes	3.32	0.95	1–5	-0.298	-0.514	0.282	0.447
Academic6	Yes	3.78	0.81	2–5	-0.689	0.249	0.321	0.434
Academic7	Yes	3.54	0.98	1–5	-0.418	-0.419	0.228	0.472
Academic8	No	3.76	0.93	1–5	-0.930	0.734	-0.100	0.600
Wellbeing1	No	3.24	0.97	1–5	-0.269	-0.520	0.296	0.702
Wellbeing2	No	3.70	0.76	2–5	-0.489	0.067	0.575	0.480
Wellbeing3	No	3.96	0.71	1–5	-0.668	1.554	0.490	0.546
Wellbeing4	No	4.22	0.74	1–5	-1.014	1.955	0.413	0.592

Note. All 169 participants had complete responses to the 28 VIS items. SD = sample standard deviation. Statistics are based on scored responses; the 11 items marked “Yes” were reverse-scored as 6 − original response. Higher scored values indicate more favorable responses (greater benefits or lower burden, as applicable).

Skewness and excess kurtosis are bias-corrected estimates; excess kurtosis is expressed relative to the normal distribution (0). Corrected item–total r is the Pearson correlation between an item and the sum of the other items in its original proposed domain. Alpha if item deleted is the unstandardized Cronbach’s alpha for the remaining items in that

domain, not for the full 28-item pool. The dash indicates that alpha is undefined after deleting one item from the two-item Physical domain.

Original-domain Cronbach's alpha: Social (8 items), 0.661; Mental (6 items), 0.579; Physical (2 items), 0.643; Academic (8 items), 0.503; Wellbeing (4 items), 0.649. These estimates describe the original item pool and should not be confused with reliability estimates for the retained 16-item instrument.
